# Designed DNA-Encoded IL-36 Gamma Acts as a Potent Molecular Adjuvant Enhancing Zika Synthetic DNA Vaccine-Induced Immunity and Protection in a Lethal Challenge Model

**DOI:** 10.3390/vaccines7020042

**Published:** 2019-05-22

**Authors:** Lumena Louis, Megan C. Wise, Hyeree Choi, Daniel O. Villarreal, Kar Muthumani, David B. Weiner

**Affiliations:** 1 Vaccine and Immunotherapy Center, The Wistar Institute, Philadelphia, PA 19104, USA; llouis@wistar.org (L.L.); hychoi@wistar.org (H.C.); kmuthumani@wistar.org (K.M.); 2 Inovio Pharmaceuticals, Plymouth Meeting, PA 19462, USA; megan.wise@inovio.com; 3 Incyte Corporation, Wilmington, DE 19803, USA; dovill33@gmail.com

**Keywords:** IL-36, adjuvant, DNA, Zika

## Abstract

Identification of novel molecular adjuvants which can boost and enhance vaccine-mediated immunity and provide dose-sparing potential against complex infectious diseases and for immunotherapy in cancer is likely to play a critical role in the next generation of vaccines. Given the number of challenging targets for which no or only partial vaccine options exist, adjuvants that can address some of these concerns are in high demand. Here, we report that a designed truncated Interleukin-36 gamma (IL-36 gamma) encoded plasmid can act as a potent adjuvant for several DNA-encoded vaccine targets including human immunodeficiency virus (HIV), influenza, and Zika in immunization models. We further show that the truncated IL-36 gamma (opt-36γt) plasmid provides improved dose sparing as it boosts immunity to a suboptimal dose of a Zika DNA vaccine, resulting in potent protection against a lethal Zika challenge.

## 1. Introduction

The most successful approach to controlling infectious diseases on a global scale has been through vaccination. Vaccines have led to control, eradication, or near eradication of several infectious diseases, positively impacting both human longevity and the quality of life. However, much work remains in this area. For many targets, current studies have suggested the need for adjuvants which can provide a number of benefits, including improved vaccine effectiveness, as discussed in several papers and reviews [1,2,3,4,5,6,7,8,9]. Adjuvants can boost overall immune responses to a specific vaccine, thereby requiring either a lower dose or fewer immunizations, improving protection and compliance as well as increasing the global vaccine supply for a particular product [3]. Adjuvants can also help skew and tailor the immune response, which may be useful in scenarios where specific correlates of protection are understood [10,11,12]. Furthermore, adjuvants can boost immunity and shorten the time to induce a protective vaccine response in populations that traditionally have a difficult time mounting protective responses, including the elderly and immunocompromised patients [3]. Alum, the most widely used adjuvant among current licensed vaccines, is well documented to enhance humoral immunity [9,13]. Newer vaccine adjuvants including MF59 and the Adjuvant Systems group 03 and 04 (AS03, AS04, respectively) have also been licensed and shown to improve antibody responses to antigens as well as provide dose-sparing effects (among other benefits) for humoral responses [14,15,16,17,18]. Shingrix, the latest vaccine approved to protect against reactivation of herpes zoster and postherpetic neuralgia (shingles), is a recombinant vaccine made of glycoprotein E and AS01_B_ adjuvant [5,19,20]. This vaccine demonstrated an efficacy of over 95% against herpes zoster, with greater efficacy compared to a live attenuated vaccine, ZostaVax, highlighting the impact that adjuvants can have on vaccine outcomes. However, in spite of this success, there is still a major need in the clinic for adjuvants that can improve cytotoxic T lymphocyte (CTL) responses [7], and there is a lot of exciting work being done in this field. Some of this work includes nontraditional adjuvants such as pathogen-recognition receptor agonists, liposomes, nanoparticles, and gene-encoded adjuvants that can potentially jumpstart the innate immune system and work in concert with the adaptive immune arm to drive lasting memory against antigen [6]. One cytokine, Interleukin-12 (IL-12), has garnered much attention in the field for its adjuvant properties in a number of preclinical models [21,22,23,24,25,26,27]. In addition, data from a clinical study showed that inclusion of plasmid IL-12 as part of a human immunodeficiency virus (HIV) synthetic DNA vaccine increased T cell magnitude and response rates in people [28]. These data encourage further investigation of additional less well-studied cytokines as DNA or other potential adjuvants to further broaden immunity and improve cellular as well as humoral immunity for DNA-encoded antigens.

The IL-36 family is made up of the pro-inflammatory mediators alpha, beta, and gamma, as well as antagonist IL-36Ra [29,30]. This relatively novel cytokine family remains poorly understood, although recent important studies have begun to shed light on their mechanism of action. The IL-36 family is a part of the IL-1 superfamily, of which alpha, beta, and gamma are agonists. Upon binding to the IL-36 receptor (IL-36R) and recruitment of the interleukin-1 receptor accessory protein (IL-1RAcP), these cytokines activate the nuclear factor-kappa-light-chain-enhancer of activated B cells (NF-κB) and mitogen-activated protein kinases (MAPK) pathway, resulting in the stimulation of pro-inflammatory intracellular responses, whereas binding of the antagonist, IL-36Ra, prevents recruitment of IL-1RAcP and does not lead to intracellular response [31,32,33]. IL-36R is primarily expressed on naïve CD4^+^ T cells, but is also found on dendritic cells, while the cytokines are expressed mainly in skin keratinocytes and epithelium, although they are also expressed at low levels in the lung, kidneys, and intestine [29,34,35,36]. Given reports of IL-36 beta’s ability to amplify Th1 responses [37,38], we sought to understand whether these cytokines could act as adjuvants for DNA vaccination models. Here, we describe that a novel designed truncated IL-36 gamma (opt-36γt), as a co-formulated adjuvant plasmid, boosts humoral as well as CD4^+^ and CD8^+^ T cell immunity against three model synthetic DNA antigens including HIV envelope (Env), Influenza hemagglutinin 1 (HA1), and Zika premembrane and envelope (prME). Furthermore, opt-36γt enhanced protection by improving both clinical symptoms and mortality against a Zika virus (ZIKV) challenge and provided significant dose sparing for the Zika vaccine as studied using a suboptimal vaccine dose model. This not only supports the potential of opt-36γt as a gene adjuvant, but also highlights an underappreciated area of importance for protective cellular immune responses in Zika virus pathogenesis. Further investigation into opt-36γt as a potential new adjuvant for enhancing immunity against vaccine antigens is warranted. 

## 2. Materials and Methods 

### 2.1. DNA Constructs

**Figure 1:**
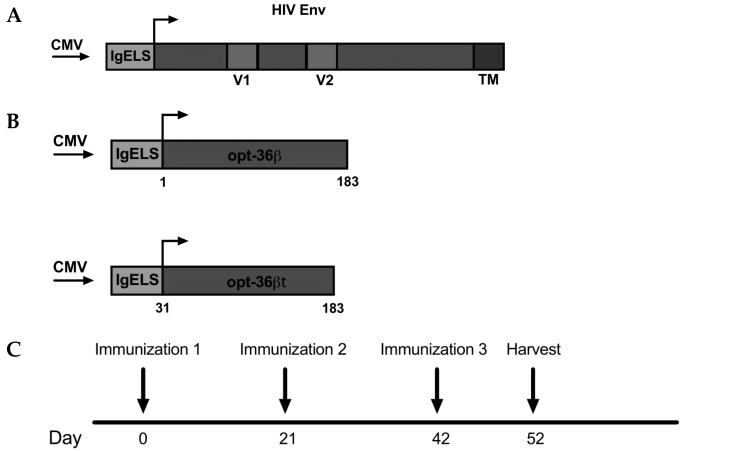

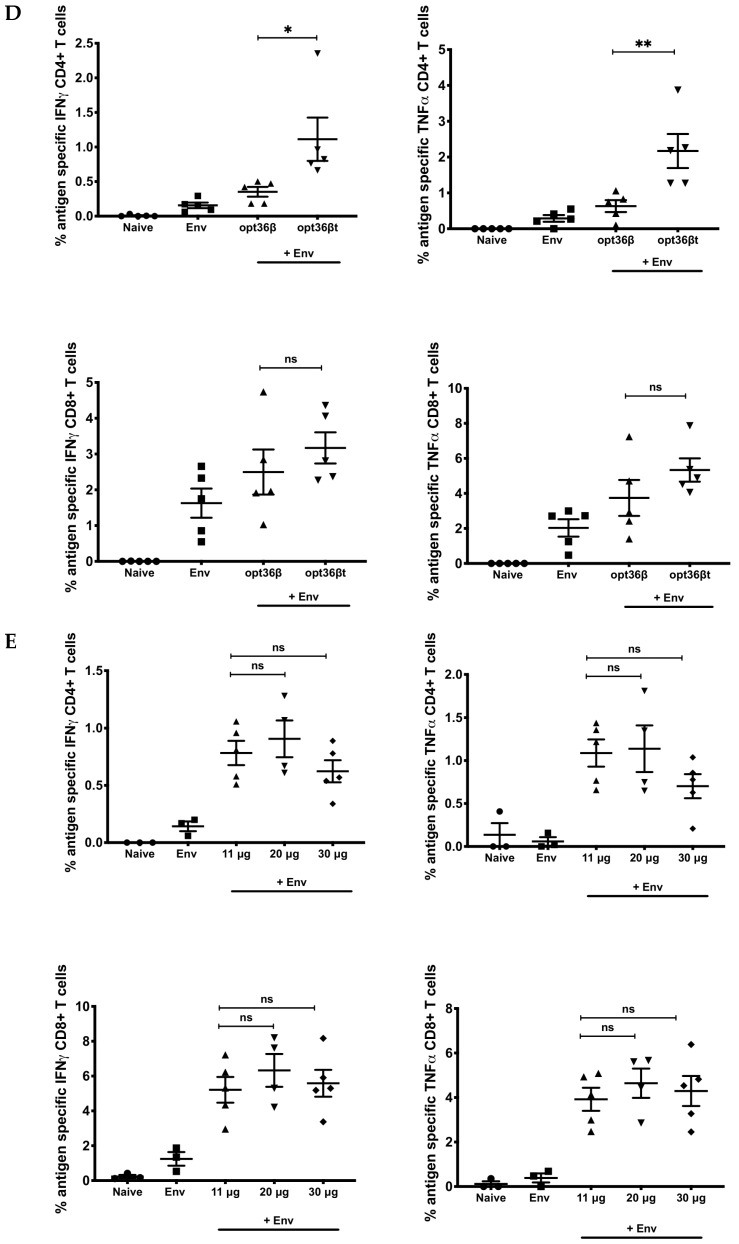
Truncation of IL-36 beta enhances immune responses to HIV Env DNA vaccine. (**A** and **B**) Map of plasmid construct design. HIV Consensus Clade C vaccine plasmid, full length IL-36 beta plasmid and IL-36 beta truncated 9 amino acids N-terminal to anchoring residue. Each construct contains a cytomegalovirus (CMV) promoter followed by an IgE leader sequence. (**C**) Immunization delivery schedule. C57BL/6 mice were immunized three times at three week intervals. (**D**) Env specific CD4^+^ and CD8^+^ T cell responses by intracellular cytokine staining after peptide stimulation. E Opt-36βt dosing study of Env specific CD4^+^ and CD8^+^ T cell responses by intracellular cytokine staining after peptide stimulation. * *p* < 0.05, ** *p* < 0.005, *** *p* < 0.0005, **** *p* < 0.0001.

The HIV consensus clade C envelope, Influenza HA1, and Zika prME DNA vaccines used in these studies are as previously described [39,40,41]. Figures 1A, 4A, and 5A have been adapted from figures from these studies.

The sequences for murine IL-36 alpha, beta, and gamma were obtained from Uniprot (Q9JLA2-1, Q9D6Z6-1, Q8R460-1). These sequences have been modified to be RNA and codon-optimized in order to exploit the host’s natural codon preference and enhance protein expression. Furthermore, a highly efficient IgE leader sequence was inserted at the 5’ end of the IL-36 gene to promote efficient secretion of the protein. These full-length optimized IL-36 cytokine plasmids are known henceforth as opt-36α, opt-36β, and opt-36γ. 

Recent work by Towne et al. has demonstrated the need for truncation of IL-36 cytokines nine amino acids N-terminal to a conserved *A*-*X*-*Asp* motif, for full activity [42]. The second set of IL-36 plasmids have been truncated according to the data presented in the paper and are henceforth known as opt-36αt, opt-36βt, and opt-36γt. All inserts were modified as previously explained above for enhanced expression and cloned into the pGX0001 backbone (Genscript, Piscataway, NJ, USA) [43].

### 2.2. Western Blot

Transfections were performed using the TurboFectin 8.0 reagent, following the manufacturer’s protocols (OriGene, Rockville, MD, USA). Briefly, U2OS cells were grown to 80% confluence in 6-well tissue culture plates and transfected with 2 μg of opt-36αt, opt-36βt, or opt-36γt. The cells were collected 2 days after transfection, washed twice with PBS and lysed with cell lysis buffer (Cell Signaling Technology, Danvers, MA, USA). Gradient (4–12%) Bis-Tris NuPAGE gels (Life Technologies, Carlsbad, CA, USA) were loaded with transfected cell lysates and transferred to polyvinylidene difluoride (PDVF) membrane. The membranes were blocked in PBS Odyssey blocking buffer (LI-COR Biosciences, Lincoln, NE, USA) for 1 h at room temperature. To detect plasmid expression, the anti-HA (A01244 Clone 5E11D8, GenScript, Piscataway, NJ, USA) antibody was diluted 1:1000 and anti–β-actin antibody diluted 1:5000 in Odyssey blocking buffer with 0.2% Tween 20 (Bio-Rad, Hercules, CA, USA) and incubated with the membranes overnight at 4 °C. The membranes were washed with PBST and then incubated with the appropriate secondary antibody (goat anti-mouse IRDye680CW; LI-COR Biosciences) at a 1:15,000 dilution in Odyssey Blocking Buffer for 1 h at room temperature. After washing, the membranes were imaged on the Odyssey infrared imager (LI-COR Biosciences).

### 2.3. Immunofluorescence Assay (IFA)

For the immunofluorescence assay, U2OS cells were grown in 6-well tissue culture plates and transfected with 2 μg of opt-36αt, opt-36βt, or opt-36γt. Two days after transfection, the cells were fixed with 4% paraformaldehyde for 15 min. Nonspecific binding was then blocked with normal goat serum diluted in PBS at room temperature for 1 h. The plates were then washed in PBS for 5 min and subsequently incubated with anti-HA antibody at a 1:1000 (mouse anti-HA, GenScript) dilution overnight at 4 °C. The plates were washed as described above and incubated with appropriate secondary antibody (goat anti-mouse IgG-AF488, Sigma, St. Louis, MO, USA) at 1:200 dilutions at room temperature for 1 h. After washing, DAPI (Millipore Sigma) was added to stain the nuclei of all cells following manufacturer’s protocol. Wells were washed and maintained in PBS, and observed under a microscope (EVOS Cell Imaging Systems; Life Technologies, Carlsbad, CA, USA).

### 2.4. Animals

All mice were housed in compliance with the NIH, the University of Pennsylvania School of Medicine and the Wistar Institutional Animal Care and Use Committee (IACUC). Six-to-eight-week-old female C57BL/6 mice were purchased from Jackson Laboratory (Bar Harbor, ME, USA). Six-to-eight-week-old female BALB/c mice were purchased from Charles River Laboratory (Wilmington, MA, USA). Five-to-six-week-old male and female Interferon-alpha/beta receptor (IFNAR)^−/−^ mice from the Mutant Mouse Resource and Research Center (MMRRC) repository–Jackson Laboratory were also housed and treated in accordance to the above parties.

### 2.5. Animal Immunizations

For HIV dosing studies, C57BL/6 mice were immunized three times at three-week intervals with either 2.5 μg of HIV Env DNA only or 2.5 μg of HIV Env DNA and 11, 20, or 30 μg of opt-36βt in a total volume of 30 μL of water. Mice were injected using the intramuscular (IM) route in the shaved tibialis anterior muscle followed by electroporation (EP) using the CELLECTRA^®^ 3P (Inovio Pharmaceuticals, Plymouth Meeting, PA, USA) as previously described [44]. For HIV plasmid comparison studies, C57BL/6 mice were immunized three times at three-week intervals with either 2.5 μg of HIV Env DNA only or 2.5 μg of HIV Env DNA and 11 μg of opt-36αt, opt-36βt, or opt-36γt in a total volume of 30 μL of water. For influenza studies, BALB/c mice were immunized two times at two-week intervals with 1 μg of HA1 DNA plasmid alone or 1 μg of HA1 DNA plasmid and 11 μg of opt-36αt, opt-36βt, or opt-36γt in a total volume of 30 μL of water delivered intramuscularly as described above. For Zika studies, IFNAR^−/−^ mice were immunized once with 0.5 μg of Zika prME alone or 0.5 μg of Zika prME and 11 μg of opt-36γt in 30 μL of water delivered intramuscularly as described above. 

### 2.6. Animal Challenge Studies

For the Zika challenge studies, IFNAR^−/−^ mice (*n* = 12–14/group) were immunized once with 0.5 μg of Zika prME vaccine or 0.5 μg of Zika prME and 11 μg of opt-36γt. The mice were challenged with 1 × 10^5^ PFU ZIKV-PR209 virus via intraperitoneal (IP) injection on day 15. Post challenge, the animals were weighed daily. In addition, they were observed for clinical signs of disease twice daily (decreased mobility; hunched posture; hind-limb knuckle walking (partial paralysis); and/or paralysis of one hind limb or both hind limbs). The criteria for euthanasia on welfare grounds consisted of 20% weight loss or prolonged paralysis in one or both hind limbs.

### 2.7. ELISpot Assay

Precoated anti-IFN-γ 96-well plates (MabTech, Cincinnati, OH, USA) were used to quantify IFN-γ responses to vaccine. 

#### 2.7.1. For HIV Studies

Spleens were isolated from C57BL/6 mice either 10 days post final vaccination for acute time points or 50 days post final vaccination for memory timepoint. Single-cell suspensions of splenocytes were made by homogenizing and processing the spleens through a 40-μm cell strainer. Cells were then re-suspended in ACK Lysing buffer (Gibco^TM^) for 5 min to lyse red blood cells before two washes with PBS and final re-suspension in RPMI complete media (RPMI 1640 + 10% FBS + 1% penicillin–streptomycin). Two hundred thousand splenocytes were added to each well and stimulated overnight at 37 °C in 5% CO_2_ with R10 (negative control), concanavalin A (3 μg/mL; positive control), or 15-mer HIV envelope clade C peptides overlapping by 11 amino acids (NIH AIDS Research & Reference Reagent Program). Peptide pools consisted of 15-mer residues overlapping by 11 amino acids, representing the entire protein consensus sequence of HIV-1 clade C, were obtained from the NIH AIDS Research and Reference Reagent Program. The Env peptides were pooled at a concentration of 2 μg/mL/peptide into four pools, and three of the four pools were used as antigens for specific stimulation of IFN-γ release. 

#### 2.7.2. For Influenza Study

Spleens were isolated from BALB/c mice 14 days post vaccination. Single-cell suspensions of splenocytes were made by homogenizing and processing the spleens through a 40-μm cell strainer. Cells were then re-suspended in ACK Lysing buffer (Gibco^TM^, Gaithersburg, MD, USA) for 5 min to lyse red blood cells before two washes with PBS and final re-suspension in RPMI complete media (RPMI 1640 + 10% FBS + 1% penicillin–streptomycin). Two hundred thousand splenocytes were added to each well and stimulated overnight at 37 °C in 5% CO_2_ with R10 (negative control), concanavalin A (3 μg/mL; positive control), or 15 mer influenza hemagglutinin peptides overlapping by 11 amino acids spanning the length of the consensus HA1 hemagglutinin protein (GenScript). The HA1 peptides were pooled at a concentration of 1 mg/mL/peptide into four pools as antigens for specific stimulation of IFN-γ release.

#### 2.7.3. For Zika Studies 

Spleens were isolated from IFNAR^−/−^ mice 14 days post-final vaccination. Single-cell suspensions of splenocytes were made by homogenizing and processing the spleens through a 40-μm cell strainer. Cells were then re-suspended in ACK Lysing buffer (Gibco^TM^) for 5 min to lyse red blood cells before two washes with PBS and final re-suspension in RPMI complete media (RPMI 1640 + 10% FBS + 1% penicillin–streptomycin). Two hundred thousand splenocytes were added to each well and stimulated overnight at 37 °C in 5% CO_2_ with R10 (negative control), concanavalin A (3 μg/mL; positive control), or 15-mer Zika peptides overlapping by 9 amino acids spanning the length of the Zika prME protein. The Zika prME peptides were pooled at a concentration of 1 mg/mL/peptide into six pools as antigens for specific stimulation of IFN-γ release. 

After 18 h of stimulation, the plates were washed and developed following manufacturer’s protocol. The plates were then rinsed with distilled water and dried at room temperature overnight. Spots were counted by an automated ELISpot reader (Cellular Technology Ltd., Shaker Heights, OH, USA).

### 2.8. Flow Cytometry: 

For intracellular cytokine staining, two million cells were stimulated in 96-well plates with overlapping peptide pools of either HIV Env, Influenza HA1, or Zika prME protein, media alone (negative control), or phorbol 12-myristate 13-acetate (PMA) and ionomycin (BD Biosciences, San Jose, CA, USA) (positive control) for 6 h at 37 °C + 5% CO_2_ in the presence of GolgiPlug and GolgiStop^TM^ (BD Biosciences, Franklin Lakes, NJ, USA). These are the same peptides pools described in the ELISpot sections. After 6 h, cells were collected and stained in FACS buffer with a panel of surface antibodies containing live dead eFluor V450, FITC anti-CD4, Alexa Fluor 700 anti-CD44, and APC-Cy7 anti-CD8 for 30 min at 4 °C. Cells were washed and then fixed with Foxp3/Transcription Factor Fixation/Permeabilization (ThermoFischer Scientific, Waltham, MA, USA) for 20 min at 4 °C. Cells were washed with Perm/Wash buffer before intracellular staining with PE-Cy7 anti-IL-2, PerCP-Cy5.5 anti-CD3ε, PE anti-TNFα, and APC anti-IFNγ for 1 h at 4 °C. Cells were then washed with Perm/Wash buffer before suspension in Perm/Wash buffer and acquisition on a BD LSRII. All results were analyzed using FlowJo^TM^ v.10.0.

### 2.9. ELISA

The HIV ELISA was performed using 1 μg/mL HIV consensus C gp120 (Immune Technology Corp., New York, NY, USA) in PBS with 0.5% Tween 20 (PBS-T). After a blocking step, serum was diluted to 1:50 and then 4-fold from there in 1% FBS in PBS-T. Each sample was run in duplicate. After a 1 h incubation, plates washed and incubated with goat anti-mouse IgG-HRP (Santa Cruz Biotechnology, Dallas, TX, USA) at a 1:5000 dilution in 1% FBS in PBS-T. Plates were then developed as described above, and the OD450 values were obtained. 

Avidity Index ELISA: The avidity of the humoral response was assessed against universal hemagglutinin at 10 days post final vaccination for influenza studies. Plates were coated with 1 μg/mL of hemagglutinin ((H1N1) (A/New Caledonia/20/99) Immune Technology Corp., New York, NY, USA) in PBS. After a blocking step, serum was diluted to 1:50 or, for the dilution curves, 1:50 and then 4-fold from there in 1% FBS in PBS-T. Each sample was run in duplicate with half of the wells treated and half left untreated. After a 1-h incubation, plates were washed five times with PBS-T. Half of the wells for each sample were incubated with denaturing reagent (8 M urea) for 5 min while the others were incubated with PBS. Plates were washed and incubated with goat anti-mouse IgG-HRP (Santa Cruz Biotechnology) at a 1:5000 dilution in 1% FBS in PBS-T. Plates were then developed as described above, and the OD450 values were obtained. The avidity index was determined by dividing the OD450 values of the treated samples by those of the untreated samples and multiplying by 100.

### 2.10. Statistical Analysis

Statistical analysis was performed using a one-way modified ANOVA with a Turkey post-hoc test for immunogenicity studies and Mantel–Cox test for challenge studies. All analysis was performed using GraphPad Prism Software (La Jolla, CA, USA). Horizontal bars represent mean with error bars expressing the standard error. *p*-Values < 0.05 were considered as statistically significant.

## 3. Results

### 3.1. Opt-36βt Co-Formulation Leads to Enhanced Immune Responses against HIV Env DNA Vaccine Compared to Opt-36β

While the IL-36 family was discovered in 1999 [29,30,34,35,45], members of this family remain poorly understood and continue to be investigated. In the initial studies of their biology, large quantities of IL-36 ligands were needed, in greater excess than those traditionally used for cytokines, to observe their activity [32,46]. With recent reports of IL-36 cytokines gaining activity after N-terminal residue truncation [42,47,48], we studied whether truncation was important for an IL-36 in vivo produced gene adjuvant to impact immune profile of DNA vaccine antigens in an HIV Env (Figure 1A) in vivo DNA vaccine model system. We chose to initially start our studies with IL-36 beta, as IL-36 beta has been reported to amplify Th1 responses [37], making it a potential cellular adjuvant candidate. We designed two DNA constructs encoding either full-length (opt-36β) or truncated (opt-36βt) IL-36 beta (Figure 1B) for these comparative studies. We added a highly efficient IgE leader sequence to both of the sequences as well as RNA and codon optimized them in order to enhance protein expression. We then immunized C57BL/6 (B6) (*n* = 5) mice with 2.5 μg of HIV Env DNA alone or with 2.5 μg of HIV Env and 11 μg opt-36β or opt-36βt, three times at three-week intervals using the 3P electrode driven by an adaptive electroporation CELLECTRA (EP) device (Figure 1C). Spleens were harvested 10 days post-final vaccination for analysis of antigen-specific responses. We observed a significant increase in the number of antigen-specific CD4^+^ T cells that secreted IFN-γ and TNF-α in the animals whose vaccine included opt-36βt compared to opt-36β (Figure 1D). There was a trend towards a similar pattern of enhancement for the antigen-induced CD8^+^ T cell responses, but in contrast to the CD4^+^ T cell responses, this did not reach significance. A dosing study was next performed, focusing primarily on T cell induction to determine the optimal dose of opt-36βt. We found no significant difference in T cell response with higher doses and, in fact, there appeared to be a trend towards decreased immune response at the 30-μg dose of opt-36βt (Figure 1E). Going forward, we maintained our established dose of 11 μg for adjuvant plasmids for the remainder of the studies.

**Figure 2:**
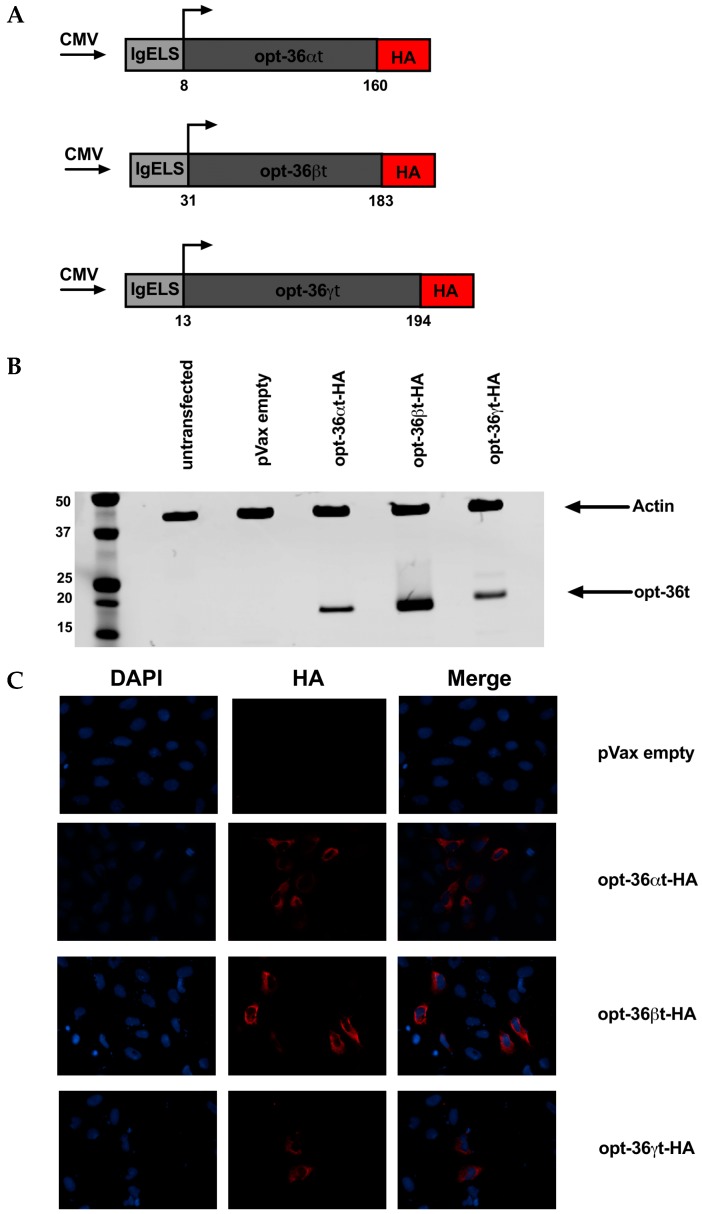
Expression of truncated IL-36 constructs. (**A**) Map of plasmid construct design for IL-36 sequences. Each sequence was truncated 9 amino acids N-terminal to conserved A-X-Asp residue. Each construct contains a cytomegalovirus (CMV) promoter followed by an IgE leader sequence besides the IL-36 sequence, and a HA tag at the C-terminus. (**B**) U2OS cells were transfected with each truncated IL-36 plasmids that contain a HA tag for detection. Lysates from these cells were used in Western blot for detection of plasmid expression. (**C**) Immunofluorescence (IFA) was performed on U2OS cells transfected with truncated IL-36 plasmids to verify plasmid expression.

Given these results, we next examined the rest of the IL-36 family as truncated cytokines. In this regard, even less is known about IL-36 alpha or gamma compared to beta, so we wanted to evaluate the immune responses in mice adjuvanted with each of the three cytokines in comparative studies. We also assessed the durability of the immune responses elicited by each of the IL-36 ligands post vaccination at a memory time point. Truncated IL-36 alpha (opt-36αt) and IL-36 gamma (opt-36γt) were designed and modified as illustrated (Figure 2A) [49,50]. An HA tag was added to the C-terminus of the sequences to facilitate in vitro detection. Construct expression in vitro was confirmed using Western blot and IFA (Figure 2B,C). 

### 3.2. Opt-36βt and opt-36γt Enhance Immune Responses against HIV Env DNA Vaccine at a Memory Time Point

**Figure 3:**
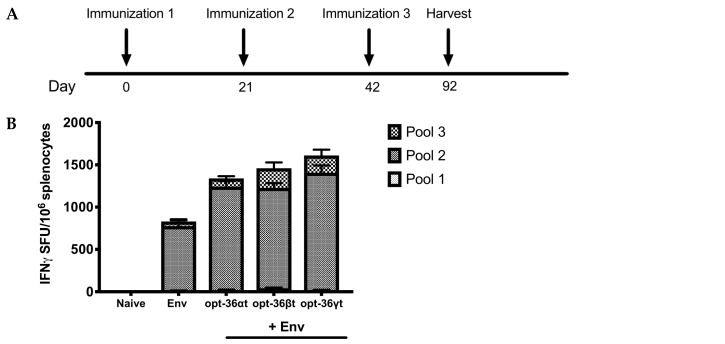

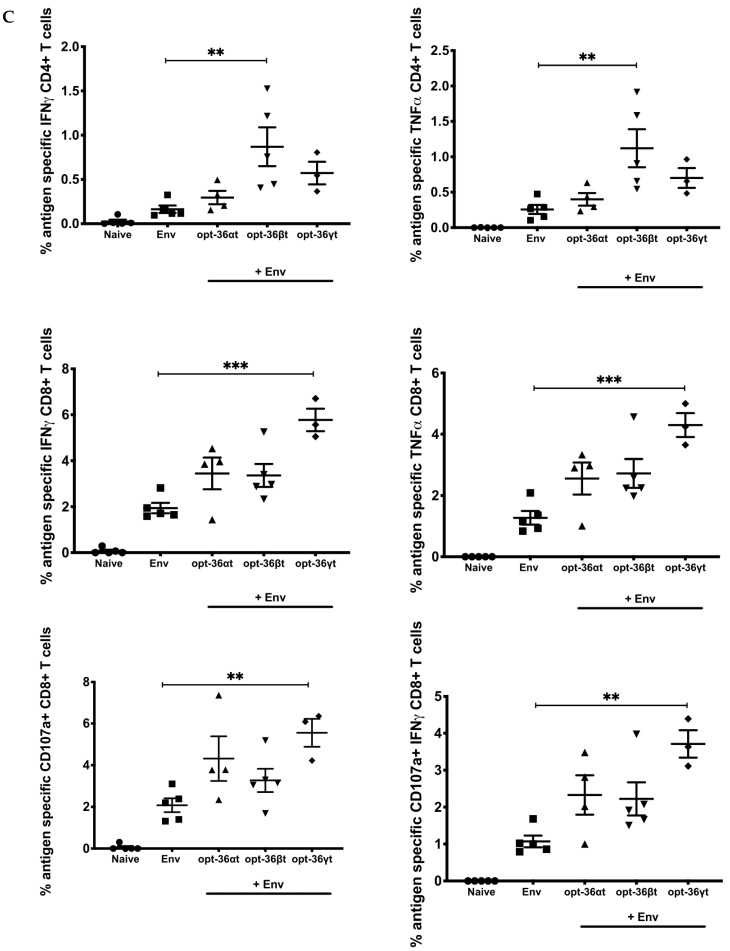
Codelivery of truncated IL-36 beta and gamma enhance immune responses against HIV Env DNA vaccine. (**A**) Immunization delivery schedule. B6 mice were immunized three times 3 weeks apart with Env alone or Env adjuvanted with the opt-36αt, opt-36βt, or opt-36γt. Sera and spleens were harvested 50 days post final vaccination to analyze antigen specific immune responses. (**B**) The frequency of Env specific IFN-γ responses (spot forming units per million splenocytes) induced after vaccination was determined by IFN-γ ELISpot assay in response to pooled Env peptides. (**C**) Env specific CD4 and CD8 T cell responses by intracellular cytokine staining after peptide stimulation. *, *p* < 0.05, ** *p*< 0.005, *** *p* < 0.0005, **** *p* < 0.0001.

A major concern in the vaccine field is the generation of vaccine candidates that can provide durable, long-term immune responses, and so we examined whether immune responses following DNA vaccination would be maintained into memory. B6 mice (*n* = 5/group) were immunized using 2.5 μg of HIV Env DNA alone or a formulation with 11 μg of opt-36αt, opt-36βt, or opt-36γt three times at three-week intervals with CELLECTRA 3P electroporation (EP) (Figure 3A). Spleens were harvested 50 days post-final vaccination to analyze antigen specific responses at a memory time point. A quantitative ELISpot was performed to determine the number of Env-specific IFN-γ secreting T cells that responded to vaccination (Figure 3B). We observed that mice immunized with the HIV vaccine alone produced an average of 775 spot-forming units (SFU)/million splenocytes, while mice adjuvanted with opt-36αt, opt-36βt, and opt-36γt had an average of 1242, 1460, and 1610 SFU/million splenocytes, respectively, supporting an enhanced response to the vaccine was driven by the adjuvants. Similar to the results observed at an acute time point, we found that mice adjuvanted with opt-36βt showed a significant increase in the percent of CD4^+^ T cells that expressed IFN-γ and TNF-α compared to vaccine only. Interestingly, mice adjuvanted with opt-36γt showed a 3-fold enhancement in the percent of antigen-specific CD8^+^ T cells which expressed IFN-γ and TNF-α (Figure 3C). We further observed that mice vaccinated with vaccine and opt-36γt had a significant increase in the percent of CD107a^+^ IFN-γ^+^ CD8^+^ T cells, suggesting the cytolytic potential of these cells (Figure 3C). We also examined the humoral response induced post vaccination, and observed that mice adjuvanted with opt-36αt and opt-36γt exhibited higher average antibody titers compared to mice immunized with Env alone, although this did not reach significance (Supplementary Materials Figure S1). Of note, mice adjuvanted with opt-36βt exhibited suppressed antibody binding compared to vaccine alone. 

### 3.3. Opt-36γt Enhances Humoral Immunity in Influenza DNA Vaccine Model

**Figure 4:**
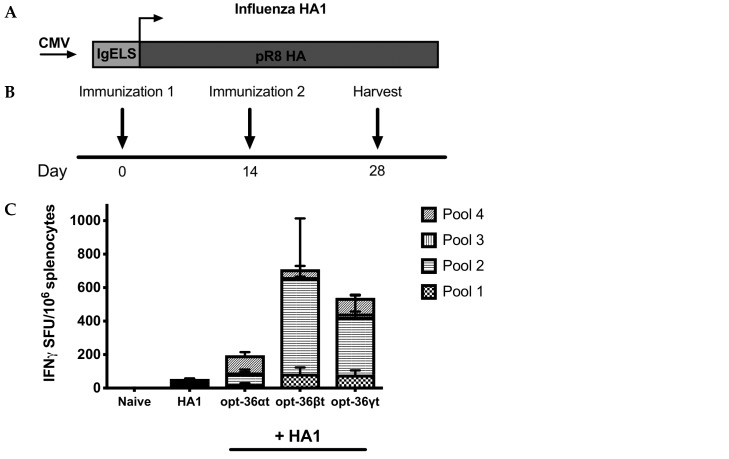

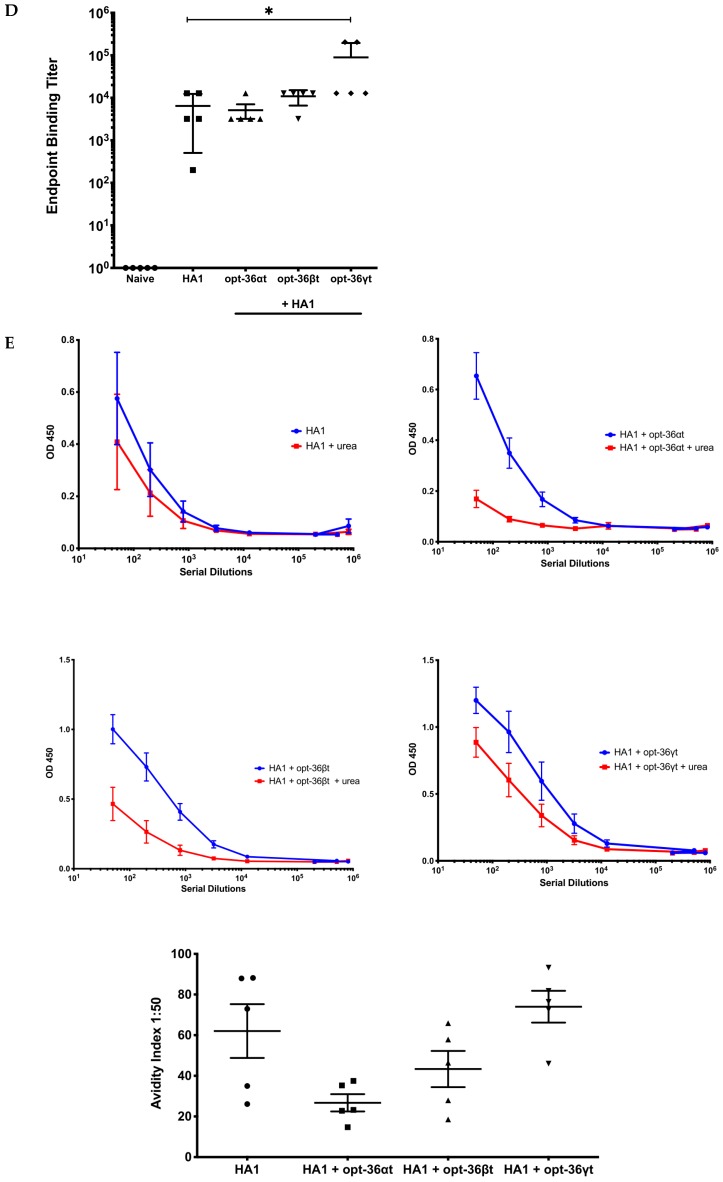
Codelivery of truncated IL-36 gamma enhances binding antibody while maintaining antibody integrity. (**A**) Map of plasmid construct design. Influenza hemagglutinin (HA) (from strain H1N1 A/PR/8/34) vaccine plasmid. Vaccine construct contains a cytomegalovirus (CMV) promoter followed by an IgE leader sequence. (**B**) Immunization delivery schedule. BALB/c mice were immunized two times two weeks apart with influenza HA1 alone or HA1 adjuvanted with opt-36αt, opt-36βt, or opt36γt. Sera and spleens were harvested two weeks post final vaccination to analyze antigen specific responses. (**C**) The frequency of HA specific IFN-γ responses (spot forming units per million splenocytes) induced after vaccination was determined by IFN-γ ELISpot assay in response to pooled HA peptides. (**D**) Endpoint binding titers post vaccination HA1 alone or HA1 + truncated IL-36 adjuvant. (**E**) The avidity of antibodies generated after vaccination at 1:50 dilution. *, *p* < 0.05, **, *p*< 0.005, ***, *p* < 0.0005, ****, *p* < 0.0001.

We next sought to extend this finding to additional antigens with a different DNA vaccine antigen. We studied opt-36αt, opt-36βt, and opt-36γt’s ability to impact immune responses driven by an HA1 Syncon influenza DNA vaccine [40]. Given the potency of the adjuvant response in the previous studies, we focused on a two-dose regimen to evaluate the vaccine-induced immune response in a dose-sparing model. BALB/c mice (*n* = 5/group) were immunized two times at two-week intervals with either 1 μg of HA1 DNA alone (Figure 4A) or 1 μg of HA1 and 11 μg of opt-36αt, opt-36βt, or opt-36γt followed by in vivo EP (Figure 4B). Ten days post final immunization, we observed both opt-36βt and opt-36γt significantly enhanced cellular responses compared to the low-dose vaccine alone (Figure 4C). We observed increased cellular responses in mice adjuvanted with opt-36αt; however, this was not as pronounced as the responses with the other two cytokines. As antibodies are known to be critical for prevention from influenza infection, we studied the binding antibodies generated post vaccination. Opt-36γt elicited significant higher endpoint binding titers compared naïve mice (Figure 4D). We further examined the quality of these antibodies by performing an ELISA based avidity test [51] to examine strength of binding to a HA1 influenza protein. Interestingly, we observed that antibodies from mice that received opt-36γt had greater antigen binding and maintained avidity compared to the antibodies from mice that received opt-36αt and opt-36βt, supporting the induction of improved magnitude of humoral responses (Figure 4E). We also examined the isotypes of the antibodies generated post vaccination, and while all immunized groups exhibited class switching, we did not observe a significant shift in IgG_1_ vs. IgG_2a_ ratios among the different groups (Supplementary Materials Figure S2). 

### 3.4. Opt-36γt Enhances Cellular Immune Responses Induced by a Zika DNA Vaccine Resulting in Enhanced Protection against Zika Challenge

**Figure 5:**
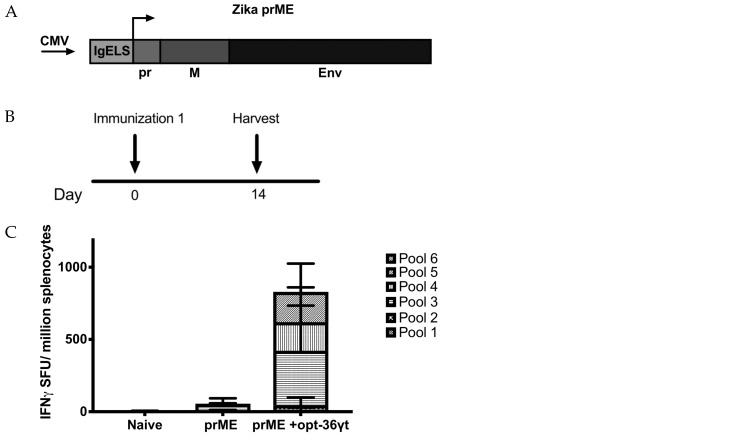

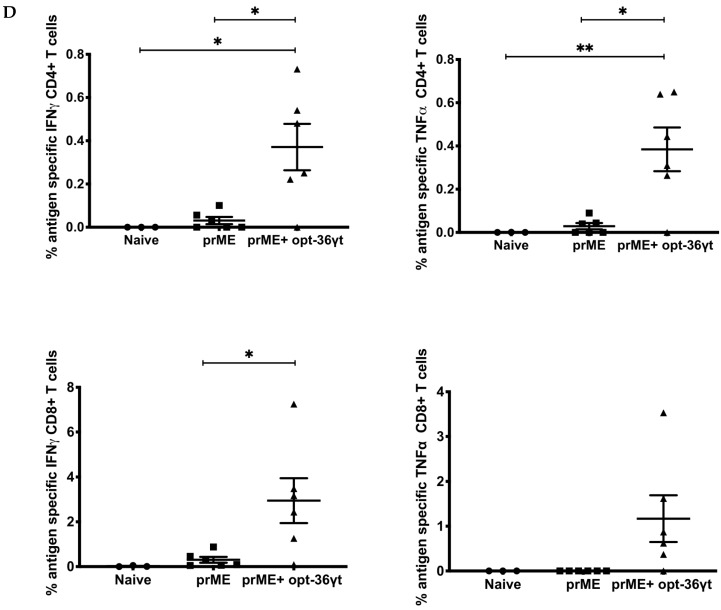
Codelivery of truncated IL-36 gamma enhances immune response to DNA prME vaccine. (**A**) Map of plasmid construct design. Consensus sequence of Zika precursor membrane and Envelope. (**B**) Immunization schedule for Zika vaccine immunization. IFNAR^−/−^ mice were immunized once either vaccine alone or vaccine + opt-36γt (*n* = 5–6 per group). Spleens were harvested two weeks post vaccination to analyze antigen specific T cell responses. (**C**) The frequency of spot forming units per million splenocytes determined by IFN-γ ELISpot assay in response to pooled Zika prME peptides. (**D**) Zika prME specific CD4 and CD8 T cell responses by intracellular cytokine staining. * *p* < 0.05, ** *p* < 0.005, *** *p* < 0.0005, **** *p* < 0.0001.

Based on the data generated in the two DNA vaccine models above, we now focused on studying opt-36γt in combination with a DNA vaccine against Zika and how vaccine-induced immune response impacted challenge outcome. This model allows us to confirm the relevance of the improved immunity and dose-sparing potential driven by the opt-36γt adjuvant. We immunized immunocompromised IFNAR^−/−^ mice (*n* = 5–6 mice/group) once with an exceptionally low dose (0.5 μg) of Zika prME DNA vaccine alone (Figure 5A) or a combination of both. Two weeks following vaccination, we harvested spleens and blood (Figure 5B). We observed that mice immunized with vaccine only did not generate significant IFN-γ ELISpot responses, but the combination of the vaccine and opt-36γt resulted in a synergy resulting in 700 SFU/million splenocytes (Figure 5C). Immunization with opt-36γt alone did not generate significant cellular responses (Figure S3). Using intracellular cytokine staining, mice adjuvanted with opt-36γt exhibited increased IFN-γ and TNF-α expressing CD4^+^ T cells as well as IFN-γ expressing CD8^+^ T cells compared to the vaccine-only treated mice (Figure 5D). Overall antibody responses were very low in all groups (Supplementary Materials Figure S4). This suggests a need for an additional vaccine boost or using higher vaccine doses to further characterize the humoral immunity induced in this model.

**Figure 6:**
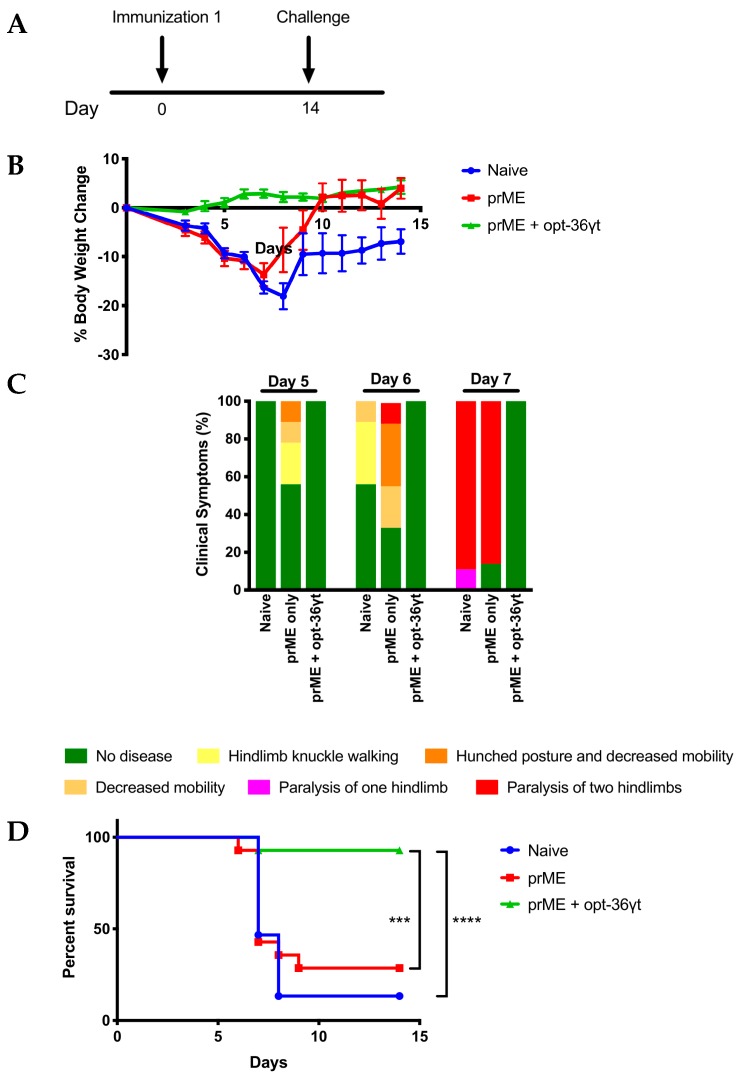
Truncated IL-36 gamma is able to protect against Zika challenge induced weight loss and mortality. (**A**) Immunization delivery schedule. IFNAR^−/−^ mice were immunized with Zika prME plasmid or prME + opt-36γt once and challenged with Zika PR209 virus two weeks later. (**B**) Mouse body weight was tracked over two-week challenge period. (**C**) Clinical symptoms of immunized mice days 5–7 post challenge. (**D**) Survival curves of mice post Zika challenge over 14 days. * *p* < 0.05, ** *p* < 0.005, *** *p* < 0.0005, **** *p* < 0.0001.

We next repeated the study and this time performed a challenge using an immunocompromised mouse challenge model, IFNAR^−/−^ mice (*n* = 12–14/ group), with a lethal dose of a validated Zika virus stock, strain PR 209. Challenge was performed two weeks after an immunization with either 0.5 μg of Zika prME alone or in combination with 11 μg of opt-36γt (Figure 6A). The animals were followed for two weeks post challenge. One of the side effects of ZIKV challenge typically observed in this mouse strain is weight loss [41]. Significant weight loss was observed in both the naïve and mice immunized with the suboptimal dose of the Zika prME vaccine alone, demonstrating substantial morbidity from the challenge (Figure 6B). Naïve mice appeared to be the most impacted, with many mice losing up to 20% of their starting body weight. The low-dose vaccine only group fared a bit better compared to the naive but still lost a considerable amount of weight. Strikingly, mice immunized with Zika prME in combination with opt-36γt were protected against weight loss, gaining weight during the course of the study. Additionally, mice were monitored for clinical symptoms during the challenge. Mice in both the naïve and vaccine-only groups became progressively sicker (i.e., hunched posture and paralysis of hind limbs) between days 5 and 7. However, the adjuvanted mice remain healthy and show no sign of disease following challenge (Figure 6C). As animals succumbed to disease they were sacrificed at predefined humane endpoints as described in the methods [41]. Mice immunized with Zika prME and opt-36γt exhibited a robust 92% survival rate, compared to 28% for mice immunized with the Zika prME only and 13% for naïve mice (Figure 6D). This data illustrates a significant benefit of the opt-36γt adjuvant in the context of this ZIKV IFNAR^−/−^ challenge model. Study in additional models is important. 

## 4. Discussion

While the IL-36 cytokine family was first discovered nearly two decades ago, it is only recently that roles for these cytokines have begun to be elucidated. The IL-36 family, members of a larger proinflammatory IL-1 family, has been primarily implicated for their potential role in pustular psoriasis and inflammation of the skin and joints [29,30,52,53]. Dysregulation of the natural IL-36 receptor antagonist or overexpression of IL-36 in the skin has been implicated in a number of skin diseases and conditions [53,54,55,56,57,58,59,60,61,62,63,64,65,66,67]. However, some of these proinflammatory properties have also piqued the scientific community’s interest regarding some of the other roles that these cytokines might play. Following reports that IL-36 beta could amplify Th1 responses in CD4^+^ T cells [37], a number of studies have shown the induction of IL-36 cytokine expression, especially IL-36 gamma, in response to infections including pneumonia, herpes simplex virus (HSV), and candidiasis [68,69,70,71,72,73], suggesting that IL-36 cytokines may play a significant role in host immunity. 

To our knowledge, this is the first study to compare the effects of all three truncated IL-36 cytokines in a vaccination model. In these studies we provide additional insight into the ability of truncated IL-36 gamma’s (opt-36γt) ability to boost immune responses using three DNA vaccine antigens. As previously demonstrated by Towne et al. [42], we found that truncation of the IL-36 cytokines’ nine amino acids at the N-terminal region was critical for their activity to enhance vaccine-induced immune responses. For future investigations of IL-36 cytokines in protective immunity studies, the truncated forms of these cytokines will almost certainly be necessary to exploit their full potential. 

In the DNA vaccine models we tested, we found that mice immunized with opt-36βt and opt-36γt were both able to enhance vaccine-induced cellular immune responses. However, where opt-36βt was able to significantly increase the number of antigen-specific IFN-γ^+^ and TNF-α^+^ CD4^+^ T cells, opt-36γt significantly increased the number of antigen-specific IFN-γ^+^, TNF-α^+^, and CD107a^+^ CD8^+^ T cells, suggesting an impact of opt-36γt to improve cytolytic activity of these cells. Further work must be done to understand the differences between the two cytokines’ seemingly preferential action on various cell compartments. Regarding humoral immunity in the influenza DNA vaccination model, we found that opt-36γt was able to increase antibody-binding titers, while opt-36βt appeared to induce antibodies that have weaker avidity. Thus, in our models, opt-36γt can improve both arms of immune response, which is likely important for many of the challenging disease targets that remain. We also found that the synergy of a non-protective dose of Zika DNA vaccine with opt-36γt was able to protect mice against a lethal ZIKV challenge, highlighting the potential of opt-36γt to affect challenge outcome and drive protection. Furthermore, opt-36γt enhanced antibody binding in both the HIV and influenza DNA models, while overall humoral responses in the Zika DNA model were lower than the other models, possibly due to the low amount of plasmid used for immunization. Other differences among the models such as mouse genotype may also be relevant and could be examined in further studies.

There is still much work to be done to fully understand the roles that the IL-36 cytokines play under both homeostatic and pathologic conditions in the host immune system. Multiple studies in mice have shown that the IL-36 cytokines may have distinct functions in response to different inflammatory stimuli. Understanding how opt-36βt and opt-36γt may exert their activities on different cell populations and against additional vaccine targets will be important for further harnessing their potential. Given their ability to enhance CD4^+^ and CD8^+^ T cell responses, opt-36γt and opt-36βt look especially promising for disease models in which cellular responses are important, such as cancer where driving CD8^+^ immunity is important to clear tumors. Studies examining the effects of opt-36γt on driving tumor-infiltrating lymphocytes (TILS) would be relevant. Work by Wang et al. has demonstrated that tumor growth was significantly inhibited in B16 melanoma IL-36 expressing cells compared to control B16 cells that did not express IL-36 gamma in mice [74]. Wang et al. also found that IL-36 gamma could promote early activation and expansion of naïve CD8^+^ T cells, in line with what we have observed in our DNA vaccine models.

Furthermore, the induction of higher binding antibodies while maintaining avidity by opt-36γt as we observed in the influenza studies may have important implications in diseases in which high avidity and affinity antibody titers are important. As more emphasis is being focused to identify immunogens that can elicit broadly neutralizing antibodies (bNabs) for HIV and influenza, adjuvants that can further refine the antibody response may prove important.

Although there appears to be a deleterious effect on skin health when IL-36 signaling is left unchecked [55], localized controlled delivery of opt-36γt as an adjuvant during intradermal vaccination could enhance vaccine responses and recruitment of cells to the site of infection. This could be especially important for infectious diseases that breach the skin’s natural barrier including herpes, malaria, and Leishmania, among others. As the largest organ in the human body, with a rich source of antigen-presenting cells (APCs) and Langerhans cells, as well as nearly 20 billion T cells, the skin is a particularly attractive site to administer an opt-36γt adjuvanted vaccine. Enhanced CTL responses in the skin can help control the spread of an infection before it is able to disseminate to other locations in the body, while greater antibody responses may help with prevention of infection. Studies that examine the delivery of opt-36γt in the skin compared to intramuscular delivery may shed light on another route to impact vaccine immune outcome as well as protection against infection.

As the global population and the demand for vaccines increase worldwide, the need to maximize immune responses while minimizing the effective dose necessary to induce protective responses will continue to grow. Here we describe the first study of an optimized plasmid encoding for a truncated form of IL-36 as a plasmid adjuvant, opt-36γt. We observed that opt-36γt exhibited a dose-sparing effect as well as enhancement of humoral and cellular immune responses to several antigens and improved challenge outcome in a well-studied mouse model system of viral challenge. Additional study of opt-36γt as a genetically encoded adjuvant is likely important.

## 5. Conclusions

As the next generation of vaccines are developed, they will likely benefit from the identification of novel adjuvants with unique immune modulating properties. Here we evaluated the adjuvant activity of 3 optimized versions IL-36 (opt-36αt, opt-36βt, and opt-36γt), novel members of the IL-1 gene family, previously reported to be involved in proinflammatory activity. We report that truncation of the IL-36 beta form (opt-36βt) enhanced immunization induced immune responses against a HIV Env DNA vaccine, compared to unadjuvanted HIV Env or the same vaccine adjuvanted by full length IL-36 beta (opt-36β). When memory responses were examined, the opt-36βt enhanced antigen specific CD4^+^ T cell responses while opt-36γt more robustly enhanced antigen specific CD8^+^ T cell responses. When these adjuvants were studied in an influenza vaccine model, opt-36γt codelivery increased antibody titers against the hemagglutinin protein. These antibody responses exhibited higher binding avidity compared to the control vaccine alone arm. We also evaluated opt-36γt’s DNA vaccine dose sparing potency in a lethal Zika vaccine challenge model. Codelivery of opt-36γt with a very low dose Zika DNA vaccine was able to potently enhance IFN-γ T cell responses resulting in potent protection against the ZIKV challenge compared to vaccine only immunized or naïve mice. This study provides proof-of-concept that an optimized plasmid encoding truncated IL-36 gamma is an important new gene adjuvant which can simultaneously enhance both humoral and cellular immunity and positively impact challenge. Further study of this promising genetic adjuvant is warranted.

## Supplementary Materials

The following are available online at https://www.mdpi.com/2076-393X/7/2/42/s1, Figure S1: (A) ELISA analysis measuring binding antibody production (measured by OD450 values) in immunized mice. The C57BL/6 mice (*n* = 5) were immunized intramuscularly three times three weeks apart with 2.5 μg of HIV Env plasmid or 2.5 μg of Env plasmid and 11 μg of opt-36αt, opt-36βt, or opt-36γt. Binding to consensus C gp120 was analyzed with sera from animals post final vaccination. (B) Average endpoint titers, Figure S2: (**A**) ELISA analysis measuring isotype binding antibody production (measured by OD 450 values) in immunized mice. BALB/c mice (*n* = 4–5) were immunized twice two weeks apart with 1 μg of HA1 DNA plasmid or HA1 DNA plasmid and 11 μg of opt-36αt, opt-36βt, or opt-36γt. Isotypes of antibodies generated were analyzed with sera from animals post final vaccination. (**B**) IgG_2a_/IgG_1_ antibody ratio was analyzed by dividing the OD450 values of IgG_2a_ by the OD450 values of IgG_1_, Figure S3: Induction of Zika specific cellular immune responses following vaccination with either Zika prME DNA vaccine alone or opt-36γt alone. ELISpot analysis measuring IFN-γ secretion in splenocytes after one immunization, Figure S4: Induction of antigen specific antibody responses following immunization with either Zika prME DNA vaccine alone or Zika prME DNA vaccine and opt-36γt after one immunization.
