# Supplementary material for: Designed DNA-Encoded IL-36 Gamma Acts as a Potent Molecular Adjuvant Enhancing Zika Synthetic DNA Vaccine-Induced Immunity and Protection in a Lethal Challenge Model
Source: Vaccines (Basel). 2019 May 22;7(2):42. doi: 10.3390/vaccines7020042 (PMC6632123; doi:10.3390/vaccines7020042)
Supplement: Supplementary file 1 [file vaccines-07-00042-s001.pdf]

vaccines

MDPI

Article

Designed DNA-Encoded IL-36 Gamma Acts As a Potent Molecular Adjuvant Enhancing Zika Synthetic DNA Vaccine-Induced Immunity and Protection in a Lethal Challenge Model

Lumena Louis [1], Megan C Wise [2], Hyeree Choi [1], Daniel O Villarreal [3], Kar Muthumani [1], and David B Weiner

Supplementary Materials

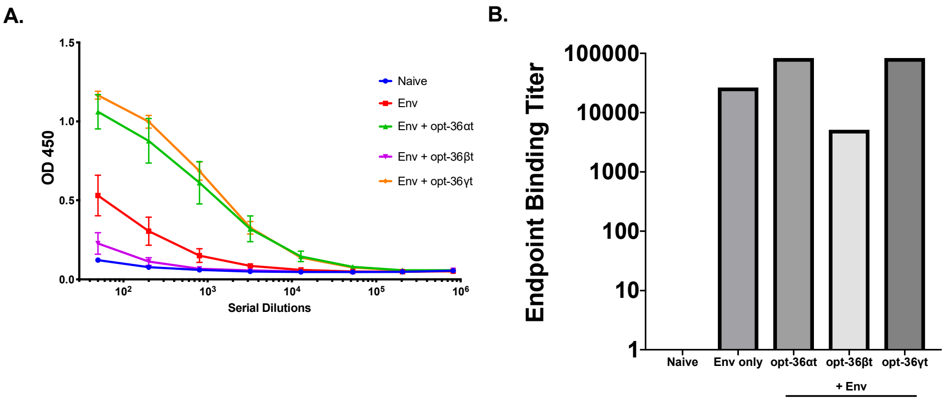

Figure S1. (**A**) ELISA analysis measuring binding antibody production (measured by OD450 values) in immunized mice. The C57BL/6 mice (n=5) were immunized intramuscularly three times three weeks apart with 2.5 μg of HIV Env plasmid or 2.5 μg of Env plasmid and 11 μg of opt-36αt, opt-36βt, or opt-36γt. Binding to consensus C gp120 was analyzed with sera from animals post final vaccination. (**B**) Average endpoint titers.

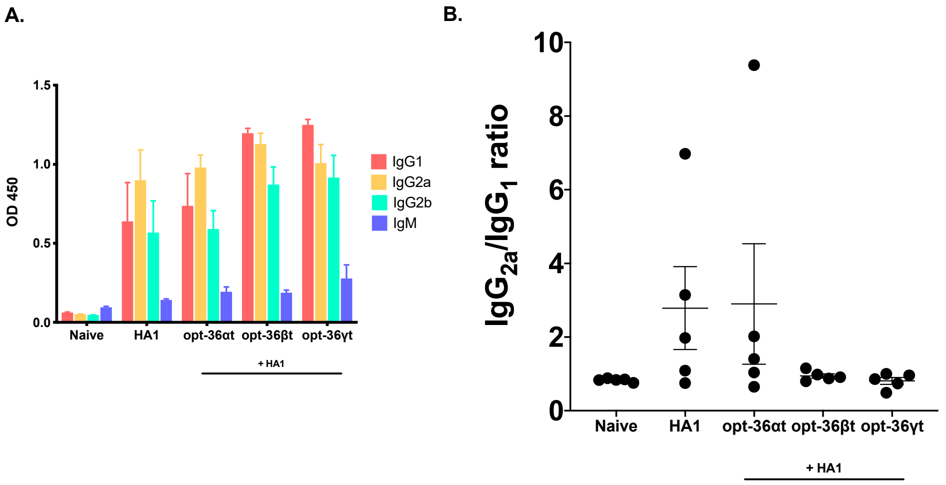

Figure S2. (**A**) ELISA analysis measuring isotype binding antibody production (measured by OD 450 values) in immunized mice. BALB/c mice (n=4-5) were immunized twice two weeks apart with 1 μg of HA1 DNA plasmid or HA1 DNA plasmid and 11 μg of opt-36αt, opt-36βt, or opt-36γt. Isotypes of antibodies generated were analyzed with sera from animals post final vaccination. (**B**) IgG_{2a}/IgG_1 antibody ratio was analyzed by dividing the OD450 values of IgG_{2a} by the OD450 values of IgG_1.

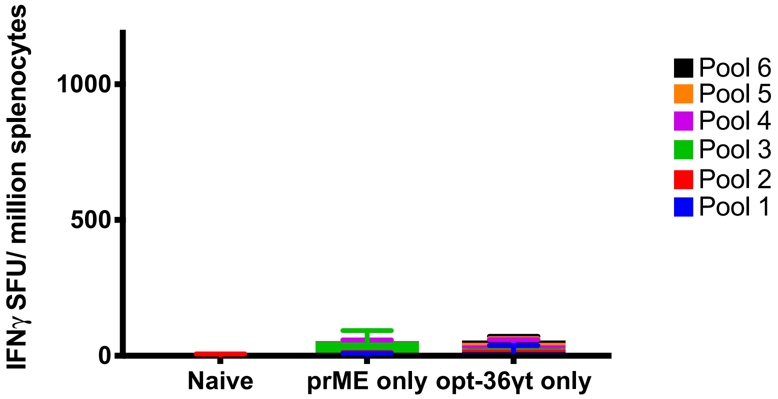

Figure S3. Induction of Zika specific cellular immune responses following vaccination with either Zika prME DNA vaccine alone or opt-36γt alone. ELISpot analysis measuring IFN-γ secretion in splenocytes after one immunization.

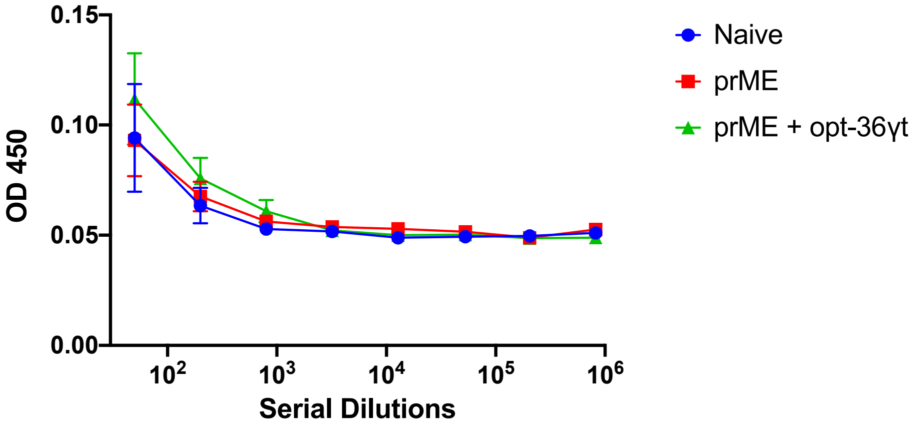

Figure S4. Induction of antigen specific antibody responses following immunization with either Zika prME DNA vaccine alone or Zika prME DNA vaccine and opt-36γt after one immunization.

© 2019 by the authors. Submitted for possible open access publication under the terms and conditions of the Creative Commons Attribution (CC BY) license (http://creativecommons.org/licenses/by/4.0/).
